# New Therapeutic Approach: Diphenyl Diselenide Reduces Mitochondrial Dysfunction in Acetaminophen-Induced Acute Liver Failure

**DOI:** 10.1371/journal.pone.0081961

**Published:** 2013-12-11

**Authors:** Nélson R. Carvalho, Edovando F. da Rosa, Michele H. da Silva, Cintia C. Tassi, Cristiane L. Dalla Corte, Sara Carbajo-Pescador, Jose L. Mauriz, Javier González-Gallego, Félix A. Soares

**Affiliations:** 1 Departamento de Química, Centro de Ciências Naturais e Exatas, Universidade Federal de Santa Maria, Campus UFSM, Santa Maria, Rio Grande do Sul, Brasil; 2 Institute of Biomedicine (IBIOMED) and Centro de Investigación Biomédica en Red de Enfermedades Hepáticas y Digestivas (CIBERehd), University of León, León, Spain; Bambino Gesu' Children Hospital, Italy

## Abstract

The acute liver failure (ALF) induced by acetaminophen (APAP) is closely related to oxidative damage and depletion of hepatic glutathione, consequently changes in cell energy metabolism and mitochondrial dysfunction have been observed after APAP overdose. Diphenyl diselenide [(PhSe)_2_], a simple organoselenium compound with antioxidant properties, previously demonstrated to confer hepatoprotection. However, little is known about the protective mechanism on mitochondria. The main objective of this study was to investigate the effects (PhSe)_2_ to reduce mitochondrial dysfunction and, secondly, compare in the liver homogenate the hepatoprotective effects of the (PhSe)_2_ to the *N-*acetylcysteine (NAC) during APAP-induced ALF to validate our model. Mice were injected intraperitoneal with APAP (600 mg/kg), (PhSe)_2_ (15.6 mg/kg), NAC (1200 mg/kg), APAP+(PhSe)_2_ or APAP+NAC, where the (PhSe)_2_ or NAC treatment were given 1 h following APAP. The liver was collected 4 h after overdose. The plasma alanine and aspartate aminotransferase activities increased after APAP administration. APAP caused a remarkable increase of oxidative stress markers (lipid peroxidation, reactive species and protein carbonylation) and decrease of the antioxidant defense in the liver homogenate and mitochondria. APAP caused a marked loss in the mitochondrial membrane potential, the mitochondrial ATPase activity, and the rate of mitochondrial oxygen consumption and increased the mitochondrial swelling. All these effects were significantly prevented by (PhSe)_2_. The effectiveness of (PhSe)_2_ was similar at a lower dose than NAC. In summary, (PhSe)_2_ provided a significant improvement to the mitochondrial redox homeostasis and the mitochondrial bioenergetics dysfunction caused by membrane permeability transition in the hepatotoxicity APAP-induced.

## Introduction

Acetaminophen (*N*-acetyl-p-aminophenol; APAP) is a drug widely employed as an analgesic and antipyretic that can induce acute liver failure (ALF) when high doses are ingested [1]. Recent data suggest a dramatic increase in ALF, liver transplants and considerable morbidity and mortality associated with APAP overdoses in the United States and many other countries [2], [3]. During overdoses, APAP is mainly metabolized in the liver by cytochrome P450, resulting in a highly reactive intermediate, N-acetyl-p-benzoquinone imine (NAPQI). NAPQI reacts directly with glutathione (GSH), causing a depletion of GSH in the liver. This redox imbalance in the liver has been shown to play a major role in ALF associated with APAP [3]. If glutathione is not replenished, NAPQI begins to form covalent bonds with cellular proteins, modifying their structure and function [4], [5]. In addition, the accumulation of neutrophils and Kuppfer cells contribute to the inflammatory process and reactive species (RS) generation in the hepatocytes [5], [6], [7]. The hepatic injury is associated with damage to subcellular organelles including mitochondria, because mitochondria are responsible for cellular energy metabolism and represent a remarkable source of intracellular RS generation in mammalian cells, effects on this organelle are critical with regard to APAP-mediated liver injuries [8].

The compound N-acetylcysteine (NAC) is the treatment of choice for acute poisoning with APAP [2]. NAC administration is beneficial for preventing or reducing ALF by increasing GSH and thiols levels, reduces the histological changes caused by oxidative stress induced by APAP overdose [1], [9], [10]. The efficacy of NAC and the prognosis are dependent on three factors, the type of APAP ingestion (acute vs. chronic), the dose of APAP ingestion and the elapsed time from APAP ingestion to the initiation of NAC treatment [9]. In clinical situations, NAC is administered after the occurrence of an APAP overdose, making the study of alternative therapies attractive.

Considering the fact that NAC efficacy is limited to a narrow window of time and situations [10], [11]. The use of organoselenium compounds could emerge as an alternative therapy. Several studies have demonstrated both the antioxidant and the anti-inflammatory properties of organoselenium compounds such as diphenyl diselenide [(PhSe)_2_] and ebselen (Ebs) [12], [13], [14]. In particular, (PhSe)_2_, the simplest of diaryl diselenides and a lipophilic organic compound of selenium, has demonstrated low toxicity in different experimental models. For example, the calculated LD_50_ in mice is 655 mg/kg when administered intraperitoneally [15]. The hepatoprotective is associated with the biochemical and pharmacological properties of the organoselenium compounds to scavenge hydrogen peroxide and other organic hydroperoxides originate from the powerful nucleophile intermediates that involve the selenol-selenolate group, which play critical roles in their glutathione peroxidase– and thioredoxin reductase–like activities [13], [15]. Earlier work from our laboratory has shown that (PhSe)_2_ is effective for the treatment of cellular damage caused by APAP [16], [17]. However our study uses for the first time the (PhSe)_2_ as a possible target to the mitochondrial dysfunction in hepatic failure caused by APAP in a new therapeutic approach.

Previously, the APAP toxicity was shown to consist of two crucial phases: the initial GSH depletion and covalent binding of NAPQI to target proteins and the subsequent increase in the mitochondrial permeability transition (MPT) and nitration of proteins [7]. In this condition, the impairment of GSH antioxidant system has been noticed to enhance the susceptibility to mitochondrial dysfunction from oxidant stress and resulting in the collapse of mitochondrial membrane potential (Δψ_m_) and ATP depletion [18]. It should be noted that MPT is mediated by oxidant stress [19]. Therefore, MPT occurs with the release of superoxide, which in turn can lead to peroxynitrite (ONOO^−^) production and tyrosine nitration, a lethal event for the cell [8]. Moreover, both oxidative damage and NAPQI have been reported to produce MPT through the oxidation of critical thiols to disulfides, which appears to be a prerequisite for membrane permeabilization [20]. Currently, it has been suggested that organoselenium present modulatory effects in relation to mitochondrial oxidative stress; however, these studies were conducted using *in vitro* models [21], [22]. Thus, there is no evidence in the literature demonstrating the effects of (PhSe)_2_ on liver mitochondrial dysfunction caused by APAP intoxication.

Thus, considering that relatively few studies have focused on the mechanisms by which these organoselenium compounds exert their pharmacological effects on APAP-induced ALF [16], [17], this study was designed to evaluate the benefits of the (PhSe)_2_ treatment under the mitochondrial dysfunction, and subsequently, compare in liver homogenate the hepatoprotective effects with *N-*acetylcysteine (NAC) during APAP-induced ALF to validate our model. This work may contribute to a better understanding of the (PhSe)_2_ mechanism of action and open new perspectives for its application.

## Materials and Methods

### Materials

(PhSe)_2_ (98%), thiobarbituric acid (TBA), 2′-7′-dichlorofluorescein (DCFH), trichloroacetic acid (TCA) and nucleotides were purchased from Sigma Chemical Co. (St. Louis, MO). All other chemicals were of analytical grade and obtained from standard commercial suppliers.

### Animals

Seven-week-old male adult Swiss albino mice (30–40 g) from our own breeding colony were used. The animals were kept on a separate animal room, on at 12 h light/dark cycle, at temperature of 22±2°C, with free access to food and water. Mice were acclimated for 7 days before initiation of any procedures. This study was approved by the Ethics and Animal Welfare Committee of Federal University of Santa Maria, Brazil.

### Experimental procedure

Briefly, the mice were randomly divided into the following groups: Control (vehicle); diphenyl diselenide [(PhSe)_2_]; N-acetylcysteine (NAC); acute liver failure (induced by APAP); acute liver failure treated with diphenyl diselenide [APAP+(PhSe)_2_] and acute liver failure treated with N-acetylcysteine (APAP+NAC). All the solutions were administered by the intraperitoneal (i.p.) route. Injections were administered at 9:00 a.m. in order to remove any confounding factors of circadian rhythm. The APAP, (PhSe)_2_ and NAC doses were described earlier [9], [16], [17]. Each group contained 7 different mice/group. Mice in the control, (PhSe)_2_ and NAC groups were injected intraperitoneally (i.p.) with saline 0.9% (20 ml/Kg), and mice in the APAP, APAP+(PhSe)_2_ and APAP+NAC groups were injected i.p. with 600 mg/Kg APAP (20 ml/Kg in saline 0.9%). One hour after saline and APAP treatment, mice were injected i.p. with 15.6 mg/Kg (PhSe)_2_ (2.5 mL/Kg in canola oil) in the (PhSe)_2_ and APAP+(PhSe)_2_. In addition, studies were done with a higher dose of NAC to validate our model. One hour after saline and APAP treatment, mice were injected i.p. with 1200 mg/kg NAC (20 ml/kg in saline 0.9%) in the NAC and APAP+NAC. The feed was available ad libitum and animals were not fasted prior to dosing. The biochemical analyses were carried out 4 h as previous studies had shown toxicity was apparent at this time [16], [17]. The animals were killed by cervical dislocation and blood was collected by cardiac puncture using heparin-rinsed 1-mL syringes (20-gauge needles) and centrifuged. The plasma was used for determination of alanine aminotransferase (ALT) and aspartate aminotransferase (AST) activities using a commercial kit (Labtest®, Diagnostica S.A., Minas Gerais, Brazil).

### Survival

For survival studies, mice were injected with 600 mg/kg and after 1 h treated with (PhSe)_2_ or NAC. Then, thirty minutes later the mice were returned to their cages and fed with food and water *ad libitum*. To determine the effect of (PhSe)_2_ and NAC on mortality of APAP-administrated mice, the survival rate after APAP administration was evaluated for 48 h.

### Liver homogenates preparation

At the end of the treatment period the liver was removed and quickly dissected, placed on ice, and immediately homogenized in cold 10 mM Tris–HCl pH 7.4. Homogenates were centrifuged at 2,000×g for 10 min to yield the low-speed supernatant fractions that were used for different biochemical assays in all trials. Besides, aliquots of liver preparations were frozen (−20°C) for posterior analysis.

### Isolation of liver mitochondria

Mice liver mitochondria were isolated at 4°C by differential centrifugation [23]. The animals were sacrificed by cervical dislocation. The livers were rapidly removed (within 1 min) and immersed in ice-cold “Ionic Medium” containing 100 mM Sucrose, 10 mM EDTA, 46 mM KCl and 100 mM Tris-HCl, pH 7.4. The tissue was minced using surgical scissors and then extensively washed. The tissue was then homogenized in a power-driven, tight-fitting Potter Elvehjem homogenizer with Teflon pestle. The resulting suspension was centrifuged for 7 min at 2,000×g in a Hitachi CR 21E centrifuge. After centrifugation, the supernatant was recentrifuged for 10 min at 12,000×g. The pellet was resuspended in “Ionic Medium+BSA” containing 100 mM sucrose, 10 mM EDTA, 46 mM KCl, 0.1% bovine serum albumin free fatty acid and 100 mM Tris-HCl, pH 7.4, and recentrifuged at 12,000×g for 10 min. The supernatant was decanted, and the final pellet was gently washed and resuspended in “Suspension Medium” containing 230 mM mannitol, 70 mM sucrose, and 20 mM Tris-HCl, pH 7.4. The pellet was washed three times with ice suspension medium buffer to get intact mitochondria, which it has been suggested that after three washings, influence of contaminating microsomes on NAD(P)H oxidation, reactive oxygen species (ROS) production and membrane permeabilization becomes negligible [24]. An aliquot of the resulting mitochondrial suspension were separated and rapidly frozen at −80°C for later biochemical analysis of GSH content, TBARS, protein carbonyls and mitochondrial enzymes.

### Measurement of lipid peroxidation (LPO)

Liver homogenate and mitochondrial membrane LPO were quantified measuring the malondiadehyde (MDA). In summary, liver homogenate and mitochondria protein were incubated in 300 µl of a medium consisting of 175 mM KCl and 10 mM Tris-HCl, pH 7.4, and then, were added to color reaction. Thiobarbituric acid reactive substances (TBARS) levels were measured at 532 nm using a standard curve of MDA [25].

### Measurement of ROS production

ROS generation was determined spectrofluorimetrically in liver homogenate and mitochondria, using H_2_DCF-DA levels as an index of the peroxide production by cellular components (1 µM) [26]. Briefly, the liver homogenate and mitochondria were added to standard medium and the fluorescence was determined at 488 nm for excitation and 525 nm for emission, with slit widths of 3 nm.

### Measurement of reduced glutathione (GSH)

GSH levels were determined in liver homogenate and mitochondria with fluorescence detection after reaction of the supernatants from deproteinized containing H_3_PO_4_/NaH_2_PO_4_–EDTA, with *O*-Phthalaldehyde (OPT) [27]. In brief, 250 mg of liver were homogenized in 3.75 mL phosphate–EDTA buffer (100 mM NaH_2_PO_4_, 5 mM EDTA, pH 8.0) plus 1 mL H_3_PO_4_ (25%), and isolated liver mitochondria (0.5 mg protein) resuspended in 1.5 mL phosphate-EDTA buffer and 500 µl H_3_PO_4_ (4.5%) were rapidly centrifuged at 100,000×g (Hitachi, TL-100 ultracentrifuge) for 30 min. For GSH determination, 100 µl of supernatant was added to 1.8 ml phosphate buffer and 100 µl OPT. After thorough mixing and incubation at room temperature for 15 min, the solution was transferred to a quartz cuvette and the fluorescence was measured at 420 and 350 nm emission and excitation wavelengths, respectively. GSH contents were determined from comparisons with a linear GSH standard curve.

### Measurement of antioxidant enzyme activities

The activities of antioxidant enzymes, total superoxide dismutase (SOD), catalase (CAT), glutathione S-transferase (GST), glutathione reductase (GR), and glutathione peroxidase (GPx) have been measured in liver homogenates.

Liver homogenate total SOD activity was measured by the capacity of inhibiting auto-oxidation of adrenaline to adrenochrome at 480 nm [28]. The liver supernatant (5 µg protein) was added to a medium containing 2 mM EDTA, 50 mM NaHCO_3_/Na_2_CO_3_ buffer (pH 10.3) and adrenaline (4 mM).

The CAT enzyme activity was determined in liver homogenate in according to the method previously proposed [29]. Liver homogenate (5 µg protein) was added to a medium containing potassium phosphate buffer (50 mM KH_2_PO_4_, 50 mM K_2_HPO_4_; pH 7.4) and H_2_O_2_ (1 mM). The kinetic analysis of CAT was started after H_2_O_2_ addition. The CAT activity was determined using the molar extinction coefficient 36 M^−1^cm^−1^ and the reaction was measured at 240 nm.

Glutathione-S-transferase (GST) activity was determined spectrophotometrically [30]. GST activity was quantified in liver homogenates (5 µg protein) in a reaction mixture containing 1 mM 1-chloro-2,4-dinitrobenzene (CDNB), and 1 mM glutathione as substrates in 0.1 M sodium phosphate buffer, pH 6.5, at 37°C. Enzyme activity was calculated by the change in the absorbance value from the slope of the initial linear portion of the absorbance time curve at 340 nm for 5 min. Enzyme activity was determined using the molar extinction coefficient 9,6 mM^−1^cm^−1^ and expressed as nmol CDNB/min/mg Prot.

Glutathione peroxidase (GPx) activity was determined spectrophotometrically at 340 nm by NADPH consumption for 2 min at 30°C [31]. The liver homogenate supernatant (5 µg protein) was added to medium containing 0.1 M phosphate buffer (0.1 M KH_2_PO_4_, 0.1 M K_2_HPO_4_ and 5 mM EDTA, pH 7.0), 1 mM GSH, 0.15 mM NADPH, 0.1 U/mL GR and 1 mM sodium azide. So, the reaction was initiated by adding the H_2_O_2_ to a final concentration of 0.4 mM. The GPx activity was determined using the molar extinction coefficient 6220 M^−1^cm^−1^ and expressed as nmol/min/mg protein.

For the measurement activity of glutathione reductase (GR) activity, the liver homogenate supernatant (5 µg protein) was added to medium containing 0.15 M phosphate buffer (0.15 M K_2_HPO_4_ and 1.5 mM EDTA, pH 7.0) and 0.15 mM NADPH. The measurements were made at 340 nm and initiated with addition of 20 mM GSSG, at 30°C for 2 min [32]. GR activity was determined using the molar extinction coefficient 6220 M^−1^ cm^−1^ and expressed as nmol/min/mg protein.

### Measurement of mitochondrial protein carbonyls

The oxidative damage to proteins was measured by the quantification of carbonyl groups based on the reaction with dinitrophenylhidrazine (DNPH) assay [33]. The mitochondria were divided into two portions containing 1 mg of protein in each. To one portion, 1 ml of 2 N HCl was added and incubated at room temperature shaking intermittently for 1 h. The other portion was treated with 1 ml of 10 mM DNPH in 2 N HCl and incubated by shaking intermittently for 1 h at room temperature. After incubation the mixture was precipitated with 10% TCA and centrifuged. The precipitate was washed thrice with 1 ml of ethanol:ethyl acetate (1∶1). The final protein precipitate was dissolved in denaturation buffer (3% SDS and 150 mM NaH_2_PO_4_; pH 6.8) and the absorption at 370 nm (DNPH-treated sample minus sample blank) was determined. Carbonyl content was calculated using the molar extinction coefficient of 22,000 M^−1^ cm^−1^ and expressed as nmol carbonyls/mg mitochondrial protein.

### Mitochondrial transmembrane electrical potential (Δψ_m_)

Mitochondrial Δψ_m_ was estimated by fluorescence changes in Safranine – *O* (10 µM) recorded by RF-5301 Shimadzu spectrofluorometer (Kyoto, Japan) operating at excitation and emission wavelengths of 495 and 586, with slit widths of 5 nm [34]. The mitochondria (0.5 mg protein) were added and 30 second latter mitochondrial respiration was induced by the addition of succinate and glutamate. Mitochondrial preparation, which was held on ice, was well maintained and did not change over the course of 5–6 hours, as determined by their ability to maintain a stable transmembrane potential in the presence of oxidizable substrates.

### Mitochondrial swelling

Measurement of mitochondrial swelling was performed in RF-5301 Shimadzu spectrofluorometer at 600 nm and slit 1.5 nm for excitation and emission. The mitochondria (0.1 mg protein) were incubated in the presence of 100 µM Ca^2+^ [19].

### Oxygen consumption of liver mitochondria

The oxygen consumption of liver mitochondria was measured using an oxymeter (Hansatech model with a Clark-type electrode) at 30°C. The cuvete containing aerated medium consisting of 225 mM mannitol, 75 mM sucrose, 10 mM KCl, 10 mM Tris-HCl, 10 mM K_2_HPO_4_, 5 mM MgCl_2_, 0.1 mM EDTA (pH 7.4) was added 0.1 mg mitochondrial protein. Pyruvate (5 mM), glutamate (5 mM) and succinate (5 mM) were placed in the medium to increase the respiratory state.

### Assessment of mitochondrial activity (MTT reduction assay)

This assay is based on the ability of mitochondrial enzymes to metabolize MTT into formazan, a reaction that takes place only in functionally intact mitochondria. The mitochondrial samples (0.1 mg protein) were incubated with 20 mM succinate at 30°C for 1 hour. After that, color was quenched with DMSO, and readings were reported as the difference in absorbance between 570 and 630 nm, and then, expressed in percent of the control [35].

### Measurement of mitochondrial antioxidant enzyme activities

The activities of antioxidant enzymes in liver mitochondria were measured by the same methods described above. The enzyme activities in isolated mitochondria were measured after disruption of mitochondria by freeze-thawing (3x), following centrifugation at 2,000xg for 1 minute at 4°C, and the mitochondrial supernatant (0.1 mg protein/mL) was add to reaction medium.

Mitochondrial MnSOD activity was measured as described previously [28]. The isolated mitochondria were assayed after incubation with 1 mM KCN. At this concentration cyanide inhibits the CuZnSOD isoform of the enzyme, but does not affect the MnSOD isoform [36].

Mitochondrial GPx activity was measured as described previously [31].

For the GR activity measurement, the mitochondria supernatant was added to reaction medium as described previously [32].

### Mitochondrial complex I and complex II assays

The samples were frozen and thawed three times, and mitochondrial electron transfer chain activity detection was performed as described below.

The activity of complex I (NADH dehydrogenase) was measured by following the oxidation of NADH [37], [38]. Approximately 0.1 mg protein of mitochondria was added to a solution containing 35 mM potassium phosphate buffer (pH 7.4) and 1.3 mM 2,6 dichloroindophenol (DCIP) in a final volume of 1 mL. The reaction was initiated with the addition of 0.15 mM NADH. Absorbance at 600 nm was monitored for 2 min to follow the rate of oxidation of NADH, and the activity was determined using an extinction coefficient of 6.22 mM^−1^ cm^−1^. After thawing, the mitochondria were found to be completely permeable to NADH.

The activity of complex II (succinate dehydrogenase) was determined by following the reduction of DCIP by succinate [39]. The reaction mixture consisted of 50 mM potassium phosphate buffer pH 7.0, 1 mM KCN, 0.05 mM DCIP, 16 mM succinate and 0.1–0.5 mg protein of mitochondrial. Absorbance changes were followed at 600 nm, using an extinction coefficient of 19.1 mM^−1^ cm^−1^ for dichloroindophenol.

### Mitochondrial ATPase activity

The mitochondrial ATPase activity was measured as the hydrolysis rate of ATP to ADP + Pi [40]. Mitochondria were incubated in buffer consisting of 50 mM Tris-HC1, pH 7.4, 75 mM KCl and 0.4 mM EDTA; 6.0 mM MgC1_2_. After pre-incubating 0.2–0.25 mg protein of mitochondrial in the reaction mixture for 2 min at 37°C, the reaction was started by adding 6.0 mM ATP and carried out for 10 min. At the end of the incubation period, the reaction was terminated by adding 0.1 ml of 5% (w/v) sodium dodecyl sulphate [41]. A control was performed in same conditions in order to obtain the non-enzymatic hydrolysis of ATP. Inorganic phosphate (Pi) production was measured using the method based on the determination of the Pi released to the reaction medium by the hydrolysis of the ATP [42]. The activity was measured spectrophotometrically at 405 nm. The values were calculated in relation to a standard curve constructed with Pi at known concentration sand also corrected by the protein content.

### Protein Determination

Protein content was determined using bovine serum albumin (BSA) as standard [43].

### Statistical analysis

Statistical analysis was performed using GraphPad (version 5.0 for Macintosh OSX, GraphPad Software, San Diego, CA). Significance was assessed by one-way analysis of variance (ANOVA), followed by Newman–Keuls's Test for post-hoc comparison. Values of *p*<0.05 were considered statistically significant.

## Results

### Effects of (PhSe)_2_ and NAC on Survival after APAP overdose

**Figure 1:**
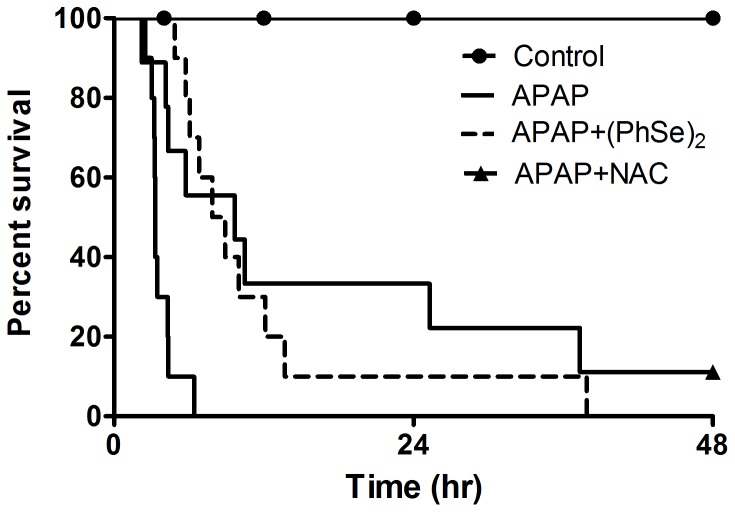
Effects of treatment with (PhSe)_2_ and NAC on the survival following lethal doses of acetaminophen. Mice were given acetaminophen (600 mg/kg, i.p.) and 1 h after were treated with or without (PhSe)_2_ (15.6 mg/kg, i.p.) and NAC (1200 mg/kg, i.p.). Survival was followed for 48 h, n = 10 per group.

Mice were monitored for 48 h to determine the effects of (PhSe)2 and NAC on the survival of mice following an APAP overdose. The mice received 600 mg/kg APAP intraperitoneally in a single dose. The APAP group mortality was pronounced when compared to the control group, which was 100% in approximately 8 h (Fig. 1). Treatment with (PhSe)2 dramatically extended the percent survival after the lethal APAP dose. All (PhSe)2 mice receiving APAP survived up to 37.5 h after treatment. A similar protection was reported following administration of NAC 1 h after the acute APAP overdose (Fig. 1). The NAC mortality was 78% compared to the control group. It is important to note that neither the (PhSe)2 and NAC controls altered the mice survival during the experimental period (data not shown).

### Effects of (PhSe)_2_ and NAC on Liver Injury Induced by APAP after 4 hours

In the present study, animals developed hepatotoxicity 4 h after a single intraperitoneal dose of 600 mg/kg APAP, as judged from the increase in plasma AST and ALT activities. In the APAP mice treated with (PhSe)_2_, the AST and ALT activity values did not significantly differ from the controls, confirming that the (PhSe)_2_ prevented the liver from APAP-induced injury (Table 1). Further studies evaluated whether the protection afforded by the (PhSe)_2_ for APAP hepatotoxicity was comparable to the NAC. The increase in plasma AST and ALT values induced by APAP was also prevented in the APAP+NAC group (Table 1). The levels of hepatotoxicity markers indicated that the (PhSe)_2_ and NAC were able to reduce the liver injury when administered following an APAP overdose (Table 1). In the present report the group treated with (PhSe)_2_ or NAC did not show hepatotoxic effects (Table 1).

**Table 1:** Effects of (PhSe)_2_ and NAC on the plasmatic transaminases levels after 4 hours.

	AST (IU/L)	ALT (IU/L)
Control	82.5±38.4	12.6±0.8
(PhSe)_2_	122.8±75.6	13.2±1.9
NAC	93.7±22.2	16.1±2.3
APAP	673.9±134.3^*^	256.2±57.1^*^
APAP+(PhSe)_2_	149.8±60.5^#^	12.0±1.1^#^
APAP+NAC	80.7±12.8^#^	19.8±6.1^#^

_2_ (15.6 mg/kg, i.p.); or NAC (1200 mg/kg, i.p.), and were killed at 4 h after the APAP treatment. Data are expressed as means ± SEM, (n = 7). Significance was assessed by one-way analysis of variance (ANOVA), followed by Newman-Keuls's Test for post hoc comparison. Significant differences are indicated by *p≤0.05 when compared with control group. Significant difference is indicated by #p≤0.05 when compared with APAP group. Mice were given acetaminophen (600 mg/kg, i.p.) and 1 h after treated with (PhSe)

### Effects of (PhSe)_2_ and NAC on Markers of the Oxidative Damage and Glutathione Redox System in Liver Homogenate following APAP Overdose

Lipid peroxidation (TBARS) caused by APAP is commonly associated with ROS generation in the liver [17]. Our results demonstrated that APAP induces a considerable increase in values of TBARS and ROS after 4 h (Table 2). Treatment with (PhSe)_2_ or NAC 1 h after the APAP dose diminished the TBARS and ROS generation to a level comparable to the control levels (Table 2). In addition, the APAP administration induced a pronounced increase in the CAT activity levels compared to the control, (PhSe)_2_ and NAC mice (Table 2), while the total SOD activity levels declined 4 h after APAP treatment (Table 2). However, the (PhSe)_2_ and NAC mice following APAP administration were able to normalize the activity levels of CAT and total SOD in the liver homogenate after 4 h (Table 2). These results suggested that (PhSe)_2_ protects against APAP toxicity by maintaining the markers of the oxidative damage at control levels, and similar levels were observed between the APAP+(PhSe)_2_ and APAP+NAC groups (Table 2).

**Table 2:** Effects of (PhSe)_2_ and NAC on oxidative damage markers in liver homogenate after 4 hours.

	TBARS (nmol MDA/mg Prot)	ROS (µmol DCF/mg Prot)	CAT (µmol H_2_O_2_/min/mg Prot)	SOD(U/mg Prot)
Control	0.3±0.10	4.1±0.7	170.3±9.9	141.2±11.4
(PhSe)_2_	0.2±0.03	4.9±1.1	186.9±21.6	171.1±15.8
NAC	0.3±0.03	3.5±0.7	149.8±24.1	144.9±9.7
APAP	1.4±0.2^*^	8.8±0.3^*^	254.4±13.9^*^	70.6±3.3^*^
APAP+(PhSe)_2_	0.8±0.1^#^	4.7±0.8^#^	190.3±21.4^#^	113.5±7.3^#^
APAP+NAC	0.5±0.1^#^	4.3±0.9^#^	170.1±29.4^#^	158.8±17.3^#^

_2_ (15.6 mg/kg, i.p.); or NAC (1200 mg/kg, i.p.), and were killed at 4 h after the APAP treatment. Data are expressed as means ± SEM, (n = 7). Significance was assessed by one-way analysis of variance (ANOVA), followed by Newman-Keuls's Test for post hoc comparison. Significant differences are indicated by *p≤0.05 when compared with control group. Significant difference is indicated by #p≤0.05 when compared with APAP group. Mice were given acetaminophen (600 mg/kg, i.p.) and 1 h after treated with (PhSe)

The glutathione redox system is a major cellular antioxidant system that combats ROS and xenobiotics in the cell. APAP depleted the liver homogenate GSH levels when compared to the control (Table 3). (PhSe)_2_ and NAC administration 1 h after the APAP dose prevented the depletion of GSH (Table 3). APAP also decreased the GPx, GR and GST activity levels, suggesting impairment in the liver homogenate redox homeostasis (Table 3). Treatment with (PhSe)_2_ following the APAP dose produced activity levels of GPx and GR that were similar to the control levels. Treatment with NAC resulted in a similar prevention of decline of GPx and GR activity (Table 3). However, the GST activity levels the APAP+(PhSe)_2_ and APAP+NAC groups remained similar to the APAP group in the liver homogenate (Table 3). Therefore, these results suggested that administration of (PhSe)_2_ 1 h after APAP treatment was sufficient to reduce the extent of the biochemical changes mediated by APAP.

**Table 3:** Effects of (PhSe)_2_ and NAC on the Glutathione redox system in liver homogenate after 4 hours.

	GSH (nmol GSH/mg Prot)	GPx (nmol NADPH/min/mg Prot)	GR (nmol NADPH/min/mg Prot)	GST (nmol CDNB/min/mg Prot)
Control	36.1±0.4	381.4±34.0	28.1±3.1	566.1±93.4
(PhSe)_2_	32.3±1.9	400.3±24.7	29.8±3.1	588.2±96.1
NAC	27.7±2.9	372.7±47.2	32.1±5.2	658.5±30.7
APAP	10.7±1.5^*^	147.7±19.1^*^	15.3±2.1^*^	298.8±20.7^*^
APAP+(PhSe)_2_	30.1±1.8^#^	365.0±37.5^#^	30.6±3.3^#^	372.7±26.3^*^
APAP+NAC	33.5±2.8^#^	335.1±32.5^#^	32.1±3.8^#^	385.2±34.6^*^

_2_ (15.6 mg/kg, i.p.); or NAC (1200 mg/kg, i.p.), and were killed at 4 h after the APAP treatment. Data are expressed as means ± SEM, (n = 7). Significance was assessed by one-way analysis of variance (ANOVA), followed by Newman-Keuls's Test for post hoc comparison. Significant differences are indicated by *p≤0.05 when compared with control group. Significant difference is indicated by #p≤0.05 when compared with APAP group. Mice were given acetaminophen (600 mg/kg, i.p.) and 1 h after treated with (PhSe)

### Effects of (PhSe)_2_ on Liver Mitochondrial Oxidative Damage and Changes in Antioxidant Enzyme Activities Induced by APAP

**Figure 2:**
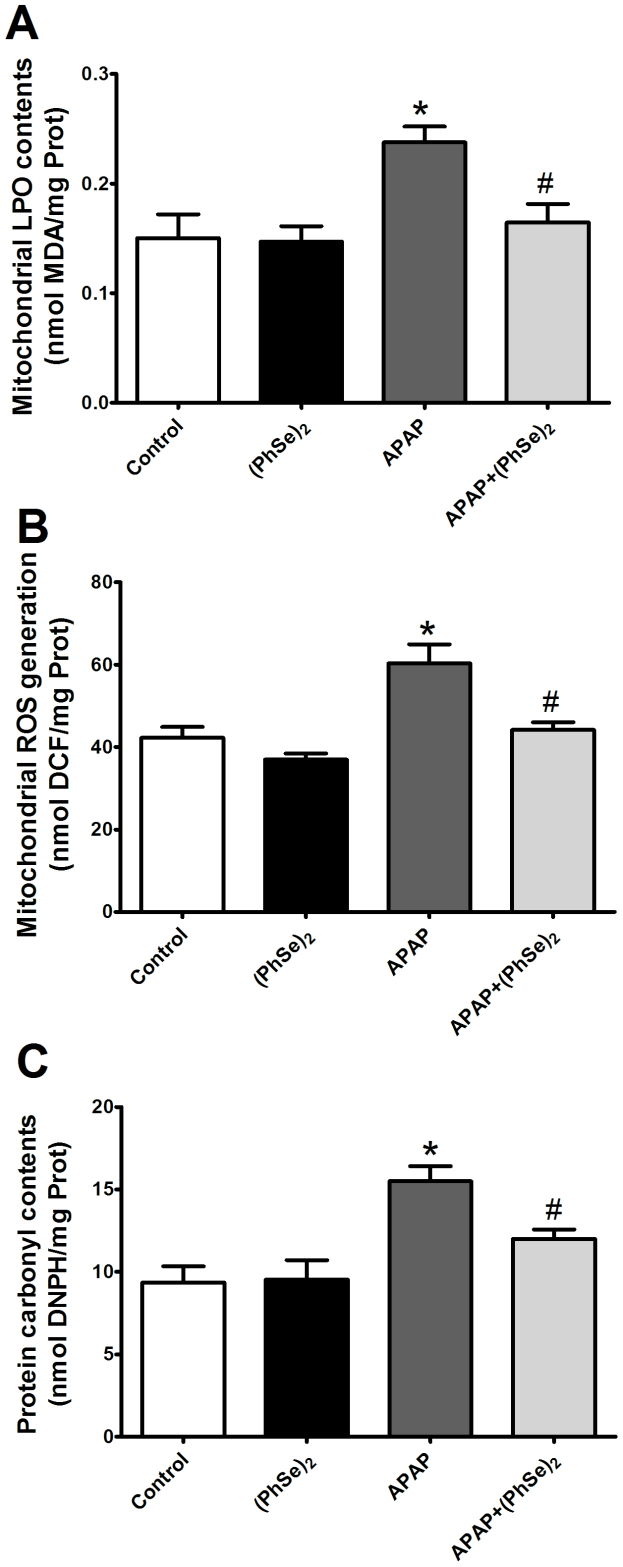
Effects of treatment with APAP and (PhSe)_2_ on oxidative damage markers in liver mitochondria of mice. (A) TBARS. (B) Oxidized H_2_DCF-DA. (C) Protein carbonyls. Mice were given acetaminophen (600 mg/kg, i.p.) and 1 h after were treated with or without (PhSe)_2_ (15.6 mg/kg, i.p.), and were killed at 4 h after the APAP treatment. Data are expressed as means ± SEM, (n = 5). Significance was assessed by one–way analysis of variance (ANOVA), followed by Newman-Keuls's test for post hoc comparison. Significant differences are indicated by ^*^p<0.05 when compared with control group. Significant difference is indicated by ^#^p<0.05 when compared with APAP group.

**Figure 3:**
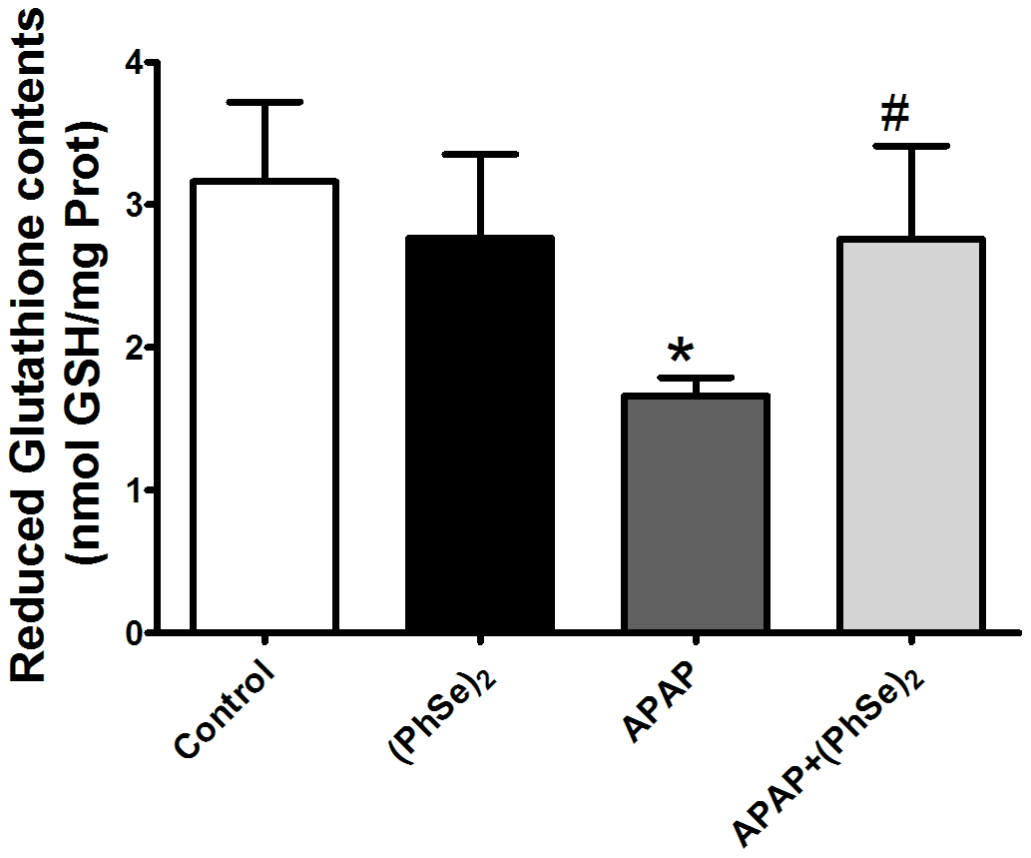
Effects of treatment with APAP and (PhSe)_2_ on reduced glutathione (GSH) levels in liver mitochondria of mice. Mice were given acetaminophen (600 mg/kg, i.p.) and 1 h after were treated with or without (PhSe)_2_ (15.6 mg/kg, i.p.), and were killed at 4 h after the APAP treatment. Dates are expressed as means ± SEM, (n = 5). Significance was assessed by one-way analysis of variance (ANOVA), followed by Newman-Keuls's test for post hoc comparison. Significant differences are indicated by ^*^p<0.05 when compared with control group. Significant difference is indicated by ^#^p<0.05 when compared with APAP group.

Oxidative stress and mitochondrial dysfunction are critical events during APAP-mediated liver injury. To investigate the effects (PhSe)_2_ on the redox balance, we measured the markers of oxidative stress and the activity of antioxidant enzymes in the liver mitochondria. The administration of APAP to the mice resulted in significantly increased levels of TBARS, protein carbonylation and an accumulation of ROS in the liver mitochondria, indicating that APAP induced oxidative stress in the liver mitochondria. The (PhSe)_2_ treatment significantly abolished all the effects in the mice exposed to APAP (Fig. 2A, 2B and 2C). The APAP overdose depleted the mitochondrial GSH levels at 4 h when compared to control levels, but administration of (PhSe)_2_ prevented the collapse of the mitochondrial glutathione redox balance caused by the APAP hepatotoxicity (Fig. 3).

**Figure 4:**
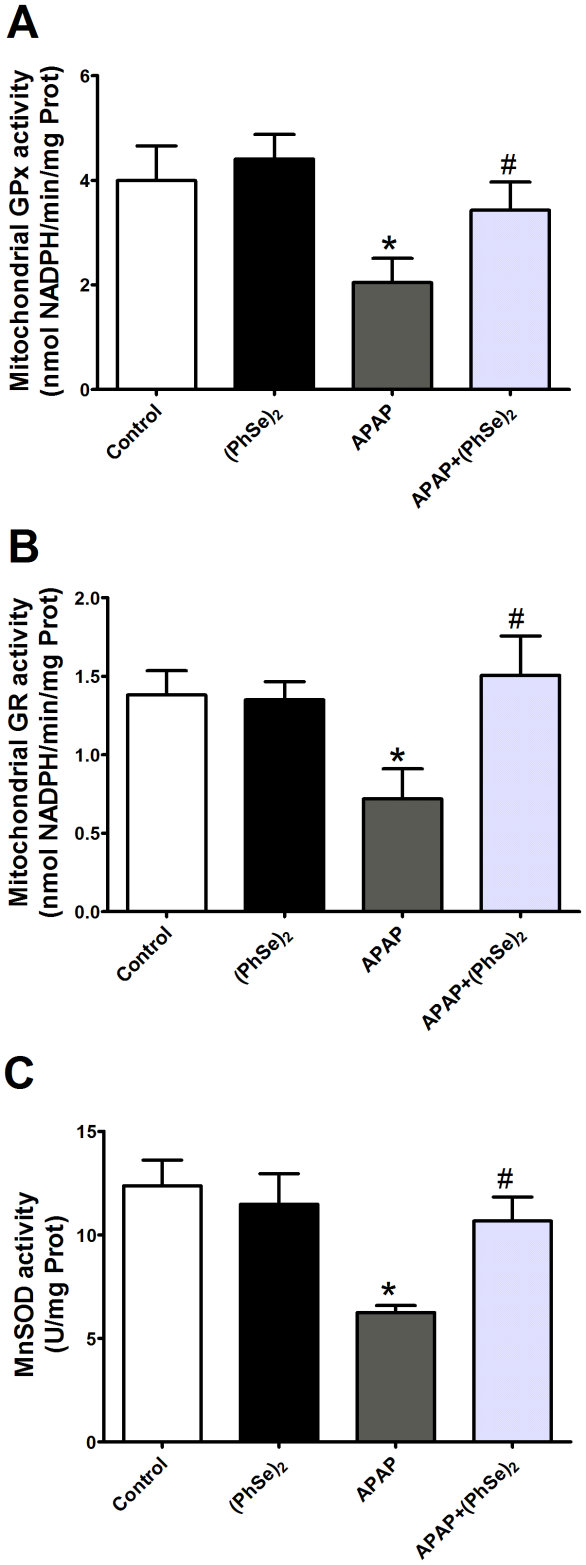
Effects of treatment with APAP and (PhSe)_2_ on antioxidant enzyme activities in liver mitochondria of mice. (A) Glutathione peroxidase (GPx) activity. (B) Glutathione reductase (GR) activity. (C) Mn Superoxide dismutase activity. Mice were given acetaminophen (600 mg/kg, i.p.) and 1 h after were treated with or without (PhSe)_2_ (15.6 mg/kg, i.p.), and were killed at 4 h after the APAP treatment. Dates are expressed as means ± SEM, (n = 5). Significance was assessed by one-way analysis of variance (ANOVA), followed by Newman-Keuls's test for post hoc comparison. Significant differences are indicated by ^*^p<0.05 when compared with control group. Significant difference is indicated by ^#^p<0.05 when compared with APAP group.

Because antioxidant enzymes contribute to the maintenance of redox equilibrium, we next measured the activity of various enzymes involved in the scavenging of ROS (GPx, GR and MnSOD) and observed that APAP administration significantly reduced the activity of the antioxidant enzymes that were analyzed in the mitochondria. The activities of the enzymes reached values that significantly differed from those in the control group. Treatment with (PhSe)_2_ prevented this outcome, and enzyme activity values in the APAP+(PhSe)_2_ group did not significantly differ from the control values (Fig. 4A, 4B and 4C). Therefore, effects of (PhSe)_2_ on the antioxidant enzyme activities contributed to the maintenance of the redox equilibrium in the liver mitochondria.

### Effects of (PhSe)_2_ on APAP-Induced Liver Mitochondrial Dysfunction

**Figure 5:**
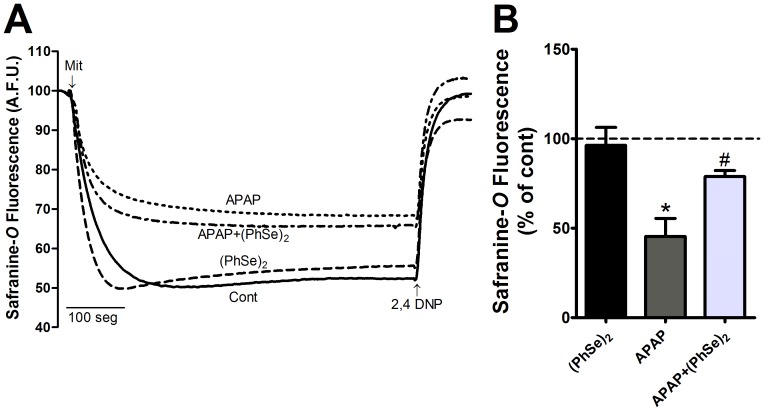
Effects of treatment with APAP and (PhSe)_2_ on the mitochondrial membrane potential in liver mitochondria of mice. (A) The traces are representative of five independent experiments. (B) Means of the five experiments mitochondrial transmembrane electrical potential (Δψ_m_). Mice were given acetaminophen (600 mg/kg, i.p.) and 1 h after were treated with or without (PhSe)_2_ (15.6 mg/kg, i.p.), and were killed at 4 h after the APAP treatment. Mitochondria (0.5 mg/ml) were incubated in the reaction medium containing 230 mM Mannitol, 70 mM Sucrose, 0.02 mM EDTA, 1 mM K_2_HPO_4_, 20 mM Tris-HCl, pH 7.4 and was energized by 5 mM Glutamate and 5 mM Succinate. The mitochondria and 2,4 DNP (100 µM) were added where indicated by arrows. Dates are expressed as means ± SEM, (n = 5). Significance was assessed by one-way analysis of variance (ANOVA), followed by Newman-Keuls's test for post hoc comparison. Significant differences are indicated by ^*^p<0.05 when compared with control group. Significant difference is indicated by ^#^p<0.05 when compared with APAP group.

**Figure 6:**
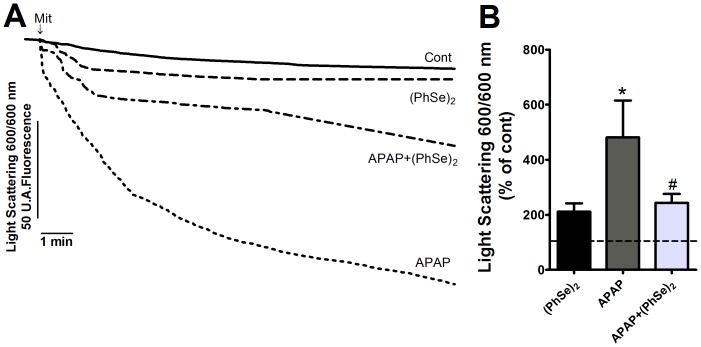
Effects of treatment with APAP and (PhSe)_2_ on PTP opening in liver mitochondria based on swelling measurements. (A) The traces are representative of five independent experiments. (B) Means of the five experiments swelling. Mice were given acetaminophen (600 mg/kg, i.p.) and 1 h after were treated with or without (PhSe)_2_ (15.6 mg/kg, i.p.), and were killed at 4 h after the APAP treatment. Mitochondria (0.1 mg/ml) were incubated in the reaction medium containing 230 mM Mannitol, 70 mM Sucrose, 1 mM K_2_HPO_4_, 20 mM Tris-HCl, pH 7.4 and was energized by 5 mM Glutamate and 5 mM Succinate. The light scattering was monitored after adding CaCl_2_ (100 µM). Dates are expressed as means ± SEM, (n = 5). Significance was assessed by one-way analysis of variance (ANOVA), followed by Newman-Keuls's test for post hoc comparison. Significant differences are indicated by ^*^p<0.05 when compared with control group. Significant difference is indicated by ^#^p<0.05 when compared with APAP group.

**Figure 7:**
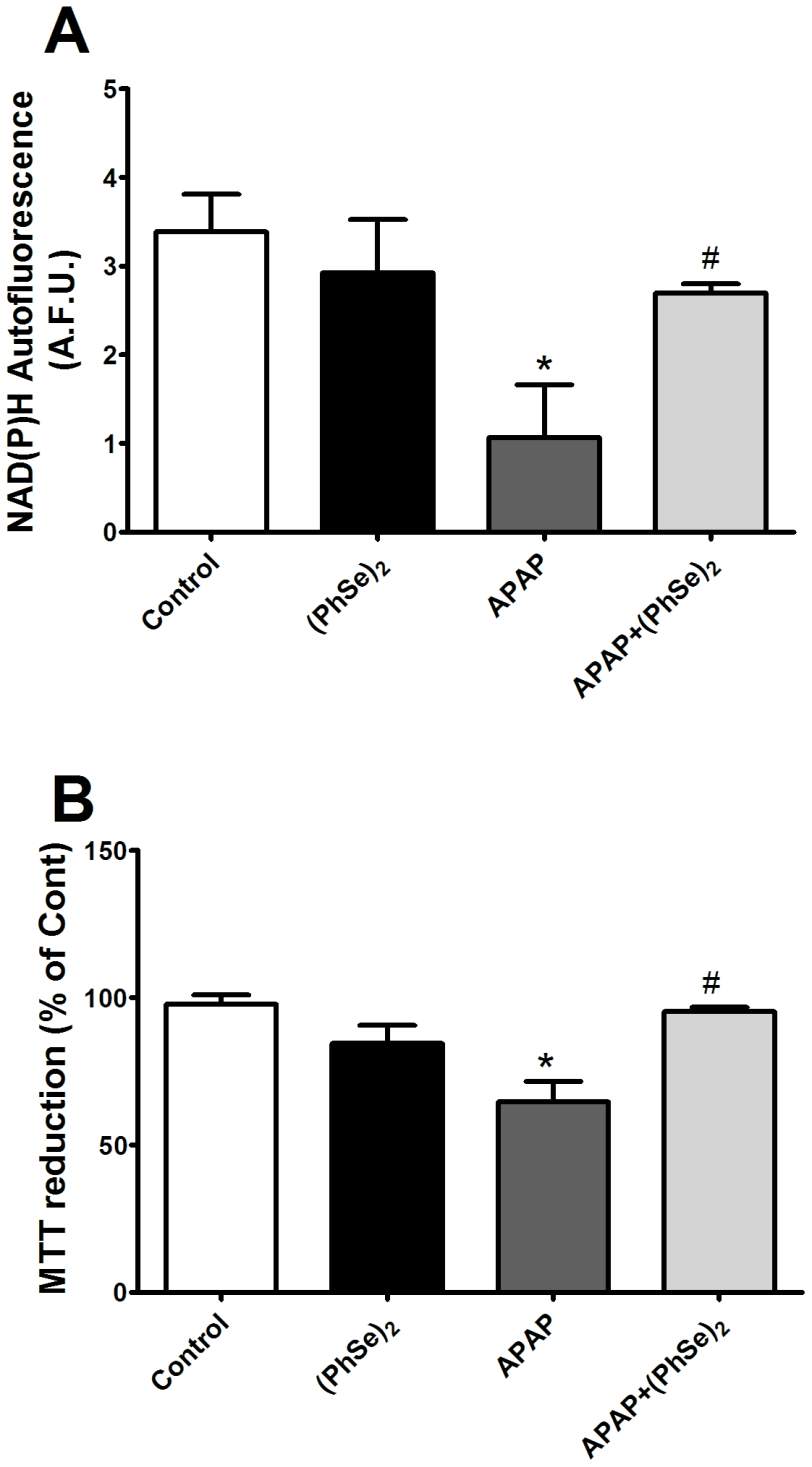
Effects of treatment with APAP and (PhSe)_2_ on mitochondria function markers in liver mitochondria of mice. (A) Pyridine nucleotide autofluorescence (NAD(P)H redox). (B) Mitochondrial activity (MTT reduction). Mice were given acetaminophen (600 mg/kg, i.p.) and 1 h after were treated with or without (PhSe)_2_ (15.6 mg/kg, i.p.), and were killed at 4 h after the APAP treatment. Dates are expressed as means ± SEM, (n = 5). Significance was assessed by one-way analysis of variance (ANOVA), followed by Newman-Keuls's test for post hoc comparison. Significant differences are indicated by ^*^p<0.05 when compared with control group. Significant difference is indicated by ^#^p<0.05 when compared with APAP group.

Next, we analyzed the effects of (PhSe)_2_ on APAP-induced liver mitochondria dysfunction. Because mitochondrial respiration and ATP production depend on the transmembrane electrical potential and mitochondrial membrane integrity, the Δψ_m_ and mitochondrial swelling were analyzed. A marked decrease of Δψ_m_ and considerable swelling were observed in the liver mitochondria of the mice exposed to APAP compared to the control group. Treatment with (PhSe)_2_ after APAP exposure prevented the loss of Δψ_m_ and prevented the mitochondrial swelling (Fig. 5 and Fig. 6, respectively). To determine whether APAP overdoses cause changes in the mitochondrial bioenergetics function, the NAD(P)H redox and mitochondrial activity were measured. APAP administration caused a significant decrease of mitochondrial NAD(P)H redox status and mitochondrial activity, but (PhSe)_2_ treatment following the APAP exposure prevented those effects (Fig. 7).

**Figure 8:**
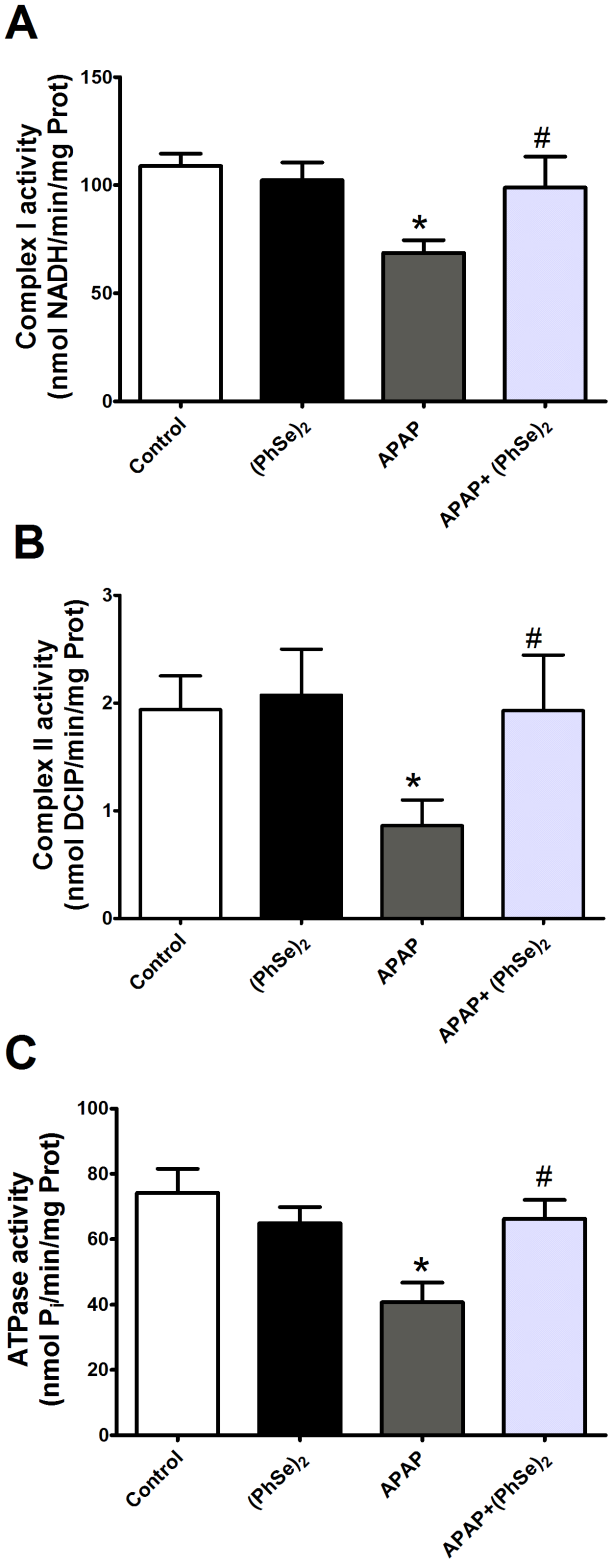
Effects of treatment with APAP and (PhSe)_2_ on the activity of respiratory chain enzymes in liver mitochondria of mice. (A) Complex I (NADH dehydrogenase) activity. (B) Complex II (succinate dehydrogenase) activity. (C) Mitochondrial ATPase activity. Mice were given acetaminophen (600 mg/kg, i.p.) and 1 h after were treated with or without (PhSe)_2_ (15.6 mg/kg, i.p.), and were killed at 4 h after the APAP treatment. Dates are expressed as means ± SEM, (n = 5). Significance was assessed by one-way analysis of variance (ANOVA), followed by Newman-Keuls's test for post hoc comparison. Significant differences are indicated by ^*^p<0.05 when compared with control group. Significant difference is indicated by ^#^p<0.05 when compared with APAP group.

To further elucidate the mechanism by which APAP impairs the mitochondrial bioenergetics function, the impact of the hepatotoxicity was assessed with regard to the electron transport chain (complex I and II) and the mitochondrial ATPase activity. The activities of complex I (NADH dehydrogenase), complex II (succinate dehydrogenase) and mitochondrial ATPase were significantly reduced in the mice with APAP-induced liver injuries, while the activity values of these enzymes did not significantly differ from the control in the APAP+(PhSe)_2_ group (Fig. 8).

Because of the observed changes in the mitochondrial electron transport chain, the mitochondrial aerobic capacity could also be affected upon exposure to APAP. Therefore, the rates of glutamate/pyruvate and succinate-supported O_2_ consumption in liver mitochondria preparations were monitored (Table 4). APAP administration caused a significant depletion of the rate of mitochondrial oxygen consumption induced for the substrates of complex I (glutamate and pyruvate) and the substrate of complex II (succinate). The treatment with (PhSe)_2_ was able to restore the oxygen consumption to values that did not significantly differ from those in the control group (Table 4). Overall, these experiments suggested that mitochondrial dysfunction plays a crucial role in mitochondrial swelling, which is consistent with the changes that were observed in the membrane potential, NADH redox state, and mitochondrial activity and O_2_ consumption, indicating that mitochondria undergo permeabilization following APAP-induced hepatotoxicity. However, the treatment with (PhSe)_2_ even after 1 h was able to reduce the mitochondrial dysfunction.

**Table 4:** Effects of treatment with (PhSe)_2_ and APAP on the respiratory rates of liver mitochondrial after 4 hours.

	Rate 1	Rate 2
	Respiration with Glut/Pyr (nmol O_2_/min/mL)	Respiration with Succ (nmol O_2_/min/mL)
Control	3.9±0.2	10.9±1.4
(PhSe)_2_	3.3±0.4	8.8±1.8
APAP	2.5±0.2^*^	5.6±1.1^*^
APAP+(PhSe)_2_	3.6±0.5^#^	8.1±1.2^#^

_2_ (15.6 mg/kg, i.p.), and were killed at 4 h after the APAP treatment. Data are expressed as means ± SEM, (n = 7). Significance was assessed by one-way analysis of variance (ANOVA), followed by Newman-Keuls's Test for post hoc comparison. Significant differences are indicated by *p≤0.05 when compared with control group. Significant difference is indicated by #p≤0.05 when compared with APAP group. Mice were given acetaminophen (600 mg/kg, i.p.) and 1 h after were treated with or without (PhSe)

## Discussion

(PhSe)_2_ delivers a hepatoprotective effect against APAP toxicity, but the mechanism remains unclear [17]. The aim of the present study was to evaluate the ability of (PhSe)_2_ to reduce the mitochondrial dysfunction and compare at the liver homogenate level the hepatoprotective effects of (PhSe)_2_ to the clinically used antidote NAC during APAP-induced ALF to validate our model.

The effects of APAP are dose dependent, with the LD_50_ estimated to be 400 mg/kg, so doses above this threshold are considered lethal [9]. After exposure to low doses of APAP, the APAP absorption is usually rapid, approximately 40–60 min, while APAP overdoses often result in slightly longer absorption times, typically within 2 h [1]. Thus, 4 h after the APAP overdose, the liver damage induced leakage of AST and ALT into the plasma, confirming that the hepatic tissue was functionally impaired when compared to those of the control, APAP+(PhSe)_2_ and APAP+NAC groups. As glucuronidation and sulfation routes become overwhelmed, the formation of NAPQI increases exponentially, with the peak levels at 4 h following the overdose [8]. Consequently, this process is followed by the perturbation of the cytosolic and mitochondrial GSH redox systems, i.e., the impairment of GSH levels and the activity of GSH-dependent enzymes (e.g., GR, GPx and GST). Additionally, the reduced activity of the total SOD and the enhanced CAT in the liver homogenate 4 h after the APAP overdose lead to a severe redox imbalance and an accumulation of RS that can exacerbate a complex cascade of reactions, culminating with lipid peroxidation and hepatocellular damage [8].

Organoselenium compounds have emerged as an alternative therapy to APAP overdoses; therefore, it is critical to establish a comparative parameter of (PhSe)_2_ and NAC, the standard clinical antidote for APAP. There are few agents similar to NAC that are able to reduce ALF when administered following an APAP overdose [5], [44]. The effectiveness of (PhSe)_2_ was similar to the classic antidote, and we observed a significant improvement in the oxidative damage markers and antioxidant enzyme activity levels in the liver homogenate. These results corroborate with the remarkable capacity of the (PhSe)_2_ to minimize all the parameters linked to oxidative stress [45]. The present study is the first to show that (PhSe)_2_ was effective at a lower dose than NAC when administered 1 h after APAP. It has been demonstrated that the selenol-selenolate intermediate group from organoselenium compounds is biochemical and physiologically more nucleophilic than the thiol-thiolate groups from cysteine residues, including from NAC [15]. Our results clearly demonstrated a depression of GST activity 4 h after the APAP overdose, and neither the (PhSe)_2_ nor the NAC treatment showed a protective effect in relation to the GST activity. The fact that the GST activity was not returned to the control level might be beneficial, as GST-null mice were previously reported to show resistance to APAP-induced hepatotoxicity [46]. Moreover, the increased activity of GR after 4 h in the APAP+(PhSe)_2_ group would serve to increase the cellular levels of GSH, which is consistent with prior results from our lab [17].

Thus, according with our results, we believed that the (PhSe)_2_ presents the therapeutic effects closely related to the three important points: maintenance of mitochondrial GSH, reduction of oxidative stress and inhibition of mitochondrial transition permeability. Firstly, the maintenance of mitochondrial GSH contributes to improve the redox homeostasis in liver, since the mitochondrial GSH pool is limited [47]. Previous reports asserted that the selective mitochondrial GSH depletion induces a significant increase of susceptibility in APAP overdose [48]. In addition, the GSH depletion precedes APAP toxicity [7]. Therefore, the concentration of intracellular GSH is a key determinant of the extent of APAP-induced hepatic injury [49]. Secondly, the abolishment of increase in oxidative markers (i.e., ROS, LPO and carbonyl proteins) is a consequence of the maintenance of antioxidant enzyme system (i.e., MnSOD, GPx and GR), which contributes to the reduction of the susceptibility to mitochondrial membrane permeability from oxidant stress [18], [50]. Decreased levels of MnSOD have been shown to significantly increase APAP toxicity, which is consistent with the generation of superoxide occurring primarily in the mitochondria with APAP toxicity [51]. Indeed, this condition could induce the mitochondrial dysfunction and mitochondrial structural degeneration [6], [7]. Finally, the inhibition of the MTP due to (PhSe)_2_ antioxidant properties that prevent a vicious cycle, which leads to a dissipation of the H^+^ gradient, impairing the oxidative phosphorylation system which is related to the bioenergetics control.

In this context, due to the (PhSe)_2_ ability to undergo oxidation–reduction cycles with concomitant scavenging of the hydroperoxides the reduction of mitochondrial oxidative damage would rescue the functionality of tricarboxilic acid cycle enzymes and the intramitochondrial redox status [52], besides, (PhSe)_2_ improved the mitochondrial antioxidant defense system and, so that can reduce the limited ability of both the H^+^ pump and bioenergetics function. Results from the present study, such as improvement of the mitochondrial bioenergetics function (Δψ_m_, mitochondrial activity and NAD(P)H redox status) and normalization of oxygen consumption at sites 1 (glutamate/pyruvate) and 2 (succinate) supports the idea of an improved energy coupling of the respiratory chain, reducing the electron escape, which reflected an improvement at the level of the oxidative phosphorylation system, substantiated by the maintenance of the mitochondrial ATPase activity. The deleterious effects on the outer and inner membrane affect the mitochondrial energy metabolism, disrupting the integrity of the respiratory chain, and induce a remarkable degree of mitochondrial swelling in the APAP group, which is consistent with the occurrence of mitochondrial membrane depolarization. One of the hallmarks of permeability transition is the exacerbated ROS generation that results in a decrease of the protein-SH and NAD(P)H redox [53].Thus, (PhSe)_2_ could reduce MPT, associated with the changes in the intramitochondrial oxidized redox state [53], [54].

Indeed, organoselenium compounds have demonstrated the ability to reduce LPO, ROS generation in the respiratory chain and the release of Fe^2+^/citrate-induced cytochrome c [21], [22]. Thus, (PhSe)_2_ exerts its effects by preserving the mitochondrial membrane integrity. Organoselenium compounds can reduce phospholipid hydroperoxides, thus protecting biomembranes from peroxidative degradation [15], consequently, causing a decrease in the collapse of Δψ_m_ and ROS production in the mitochondrial respiratory chain, which act as negative modulators of the MPT. The mechanism of action involved in the hepatoprotective effect of (PhSe)_2_ is related to its thiol peroxidase-like activity, i.e., its ability to react with peroxide after its transformation to the selenol-selenolate intermediate via either a direct interaction with GSH or another reducing thiol or by its reduction via NADPH-catalyzed thioredoxin reductase activity [55], [56]. The mitochondrial dysfunction is a consequence during the ALF induced by APAP and there is an interrelationship between the oxidative stress and MPT pore opening caused by intoxicant agents, which together can deplete NAD(P)H and affect the GSH redox status and cause a loss of Δψ_m_ [18], [57]. Additionally, the (PhSe)_2_ treatment displayed a remarkable maintenance of redox balance as well as antioxidant enzyme function, since the redox imbalance is related to the control of cell death [51], and posing a threat for both the mitochondria and the cell, with severe consequences for the proper function of organs and consequently the organism.

Notably, the treatment with (PhSe)_2_ enhances survival, extending the therapeutic window for chemical intervention. Our results demonstrate a remarkable effect extending the survival after APAP administration from 8 to 37.5 h. In line with our results, the (PhSe)_2_ administration prevents the secondary toxic effects of APAP metabolism, delaying the onset of toxic phase. Previous studies have shown that the organoselenium compounds cause a partial inhibition of cytochrome P450 [58], [59]. (PhSe)_2_ inhibited *in vitro* cytochrome P450 metabolism in rat microsomes and the IC_50_ was reported as 78 µM for microsomal activity inhibition [59]. However, another elegant study demonstrated that the ebselen presented protective effect when co-treated with APAP in hepatocytes, and this condition was probably not caused by direct reaction with APAP or inhibition of cytochrome P450 but by reduction of NAPQI by selenol intermediate [60]. Since (PhSe)_2_ shares with ebselen some chemical properties and has about twofold greater glutathione peroxidase–like activity and is also less toxic to rodents than ebselen, so, it is reasonable to suggest the formation of powerful nucleophile selenol-selenolate intermediate following by fast reduction of NAPQI to APAP, the (PhSe)_2_ could be interfering with NAPQI formation, which reduces the toxicity, and then, increasing the urinary excretion of the APAP-glucuronide metabolite. In according to Li *et al.*, selenol-selenolate intermediate was much more a reductant than a nucleophile towards NAPQI when compared with GSH [60]. It has been demonstrated that sodium selenite protected via enhanced glucuronidation of APAP thereby diverting the amount of APAP converted to NAPQI [61].

In summary, our study is the first to compare (PhSe)_2_ with NAC with regard to effectiveness as an antidote for APAP toxicity. (PhSe)_2_ was effective at a lower dose than NAC when administered 1 h after APAP. Data from the present research indicate that (PhSe)_2_ administration delayed the onset of the toxic phase, reducing APAP-induced mitochondrial dysfunction in mice and suggesting that the beneficial effects of the organoselenium treatment resulted from its antioxidant properties. The (PhSe)_2_ significantly improved the cellular and mitochondrial redox homeostasis and reduced the mitochondrial bioenergetics dysfunction caused by membrane permeability transition associated with APAP overdose. These results may help to better understand the role of mitochondrial dysfunction in APAP hepatotoxicity and support the possibility that organoselenium (PhSe)_2_ could be used as an adjuvant therapy to protect the liver from APAP-induced injuries.
